# Radiomic Cardiac MRI Signatures for Predicting Ventricular Arrhythmias in Patients With Nonischemic Dilated Cardiomyopathy

**DOI:** 10.1016/j.jacadv.2025.101684

**Published:** 2025-03-23

**Authors:** Amine Amyar, Danah Al-Deiri, Jakub Sroubek, Alan Kiang, Fahime Ghanbari, Shiro Nakamori, Jennifer Rodriguez, Daniel B. Kramer, Warren J. Manning, Deborah Kwon, Reza Nezafat

**Affiliations:** a Departments of Medicine (Cardiovascular Division), Beth Israel Deaconess Medical Center and Harvard Medical School, Boston, Massachusetts, USA; b Heart, Vascular, and Thoracic Institute, Cleveland Clinic, Cleveland, Ohio, USA; c Departments of Radiology, Beth Israel Deaconess Medical Center and Harvard Medical School, Boston, Massachusetts, USA

**Keywords:** cardiovascular magnetic resonance, implantable cardioverter-defibrillators, late gadolinium enhancement, nonischemic cardiomyopathy, radiomics, ventricular arrhythmia

## Abstract

**Background:**

Risk stratification in patients with nonischemic dilated cardiomyopathy (DCM) remains challenging. Although late gadolinium enhancement (LGE) cardiovascular magnetic resonance is recognized as a major risk factor for ventricular tachycardia/ventricular fibrillation (VT/VF), the prognostic value of LGE radiomics is unknown.

**Objectives:**

The purpose of this study was to investigate if radiomic analysis of LGE images can improve arrhythmia risk stratification in patients with DCM beyond current clinical and imaging markers.

**Methods:**

In a 2-center retrospective study, patients with DCM were identified among those who received primary prevention implantable cardioverter-defibrillators (ICDs) according to the clinical guidelines and had a cardiovascular magnetic resonance before ICD implantation. The study included patients with DCM from the Cleveland Clinic Foundation for model development and patients with DCM from Beth Israel Deaconess Medical Center for external validation. Left ventricular myocardial radiomic features were extracted from LGE images. The primary outcome was appropriate ICD intervention defined as shock or antitachycardia pacing for VT/VF. Consensus clustering and pairwise correlation were used to identify the radiomic signature. To assess the prognostic value of LGE radiomics, we built 2 logistic regression models using the development data: 1) model 1, including clinical risk factors and scar presence and 2) model 2, which combines model 1 and LGE radiomics.

**Results:**

In total, 270 patients with DCM (61% male, age 58 ± 13 years) in development data and 113 patients with DCM (71% male, age 55 ± 14 years) in external validation were included. VT/VF occurred in 40 (15%) patients in development and 16 (15%) in external validation cohorts over a median follow-up period of 4.0 (IQR: 2.5-6.1) and 2.6 (IQR: 1.2-4.1) years, respectively. Consensus clustering and pairwise correlation revealed 3 distinct radiomic features. Model 2 showed a higher C-statistic than model 1 (0.71 [95% CI: 0.62-0.80] vs 0.61 [95% CI: 0.53-0.71]; *P* = 0.028 in development and 0.70 [95% CI: 0.59-0.85] vs 0.61 [95% CI: 0.46-0.77]; *P* = 0.025 in external validation). This also significantly improved risk stratification with a continuous net reclassification index of 0.60 [95% CI: 0.29-0.91; *P* < 0.001] in development and of 0.29 [95% CI: 0.26-0.56; *P* = 0.03] in external validation. Additionally, 1 radiomic feature, namely the gray level co-occurrence matrix autocorrelation, was an independent predictor of VT/VF in both development (HR: 1.45 [95% CI: 1.10-1.91]; *P* = 0.01) and in external validation (HR: 2.38 [95% CI: 1.28-4.42]; *P* = 0.01).

**Conclusions:**

In this proof-of-concept study, radiomic analysis of LGE images provides additional prognostic value beyond LGE presence in predicting arrhythmia in patients with DCM.

Sudden cardiac death due to ventricular tachycardia or ventricular fibrillation (VT/VF) remains a major contributor to mortality in patients with nonischemic dilated cardiomyopathy (DCM).^1^ While implantable cardioverter-defibrillators (ICDs) offer a potential lifesaving intervention for those experiencing VT/VF,^2^, ^3^, ^4^, ^5^ the clinical challenge persists in determining which patients warrant such therapy for primary prevention,^3^^,^^6^ thereby posing a significant difficulty in clinical practice. Current guidelines for using ICDs as primary prevention mainly focus on reduced left ventricular ejection fraction (LVEF).^2^ However, only a minority of ICD recipients receive appropriate therapy, resulting in increased societal costs and patient morbidity. Therefore, there is an unmet need for an improved risk stratification model to identify primary prevention patients most likely to benefit from ICD intervention. This would spare patients unlikely to benefit from unnecessary morbidity and reduce the societal burden.

Cardiovascular magnetic resonance (CMR) enables noninvasive tissue characterization of the heart.^7^ Late gadolinium enhancement (LGE) is the gold standard for myocardial scar assessment.^8^ Quantitative analysis of myocardial scar burden has revealed significant associations with major arrhythmic events in patients with ischemic cardiomyopathy (ICM).^9^ In nonischemic patients, LGE has been associated with a higher risk of developing ventricular arrhythmias.^10^, ^11^, ^12^, ^13^, ^14^ Recent studies reported the potential for using myocardial LGE texture features in predicting the risk of sudden cardiac death in ICM and hypertrophic cardiomyopathy.^15^^,^^16^ A more heterogeneous scar is more likely to impact electrical conduction than a dense scar, consistent with the pathophysiology of VT, particularly in ischemic scar.^17^ However, very little is known about the impact of heterogeneous scars in idiopathic DCM and its relationship with VT/VF. Radiomic analysis of LGE can be used to objectively quantify the degree of scar heterogeneity, which could be associated with a higher risk of developing arrhythmia.

This study aimed to investigate whether radiomic analysis of LGE images can improve risk stratification in patients with DCM eligible for primary prevention ICD implantation. First, we extracted radiomic features from LGE images of the left ventricular (LV) myocardium. Then, we employed conventional statistical analysis to assess the incremental prognostic value of these radiomic features beyond the existing clinical and LGE models. Finally, we validated the radiomic signature using an external data set (Central Illustration).

**Central Illustration:**
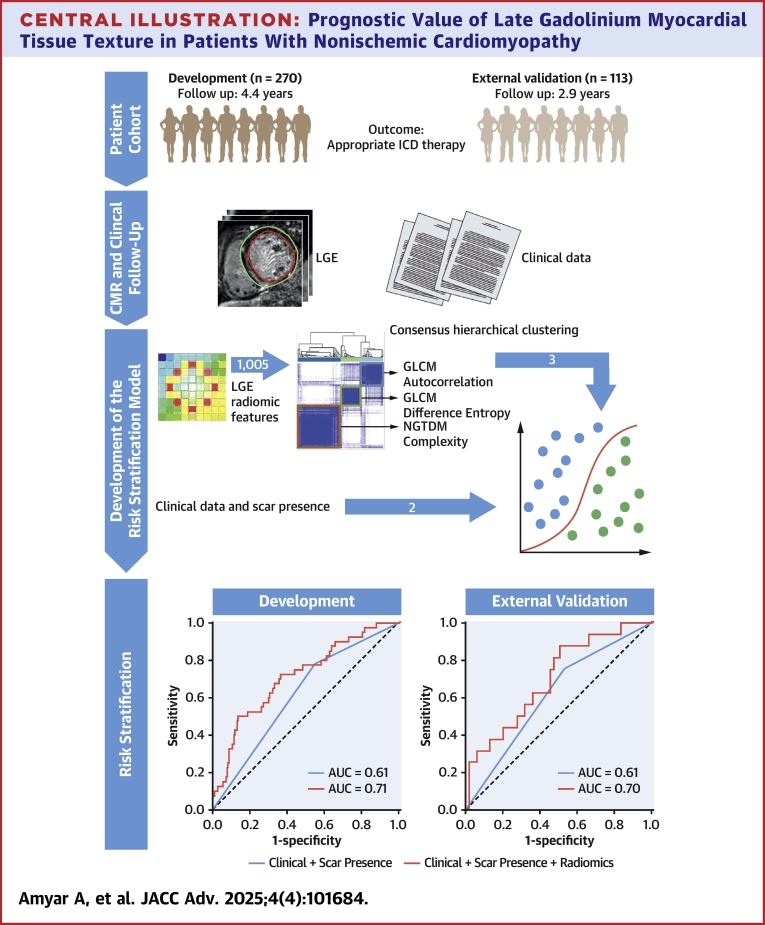
Prognostic Value of Late Gadolinium Myocardial Tissue Texture in Patients With Nonischemic Cardiomyopathy AUC = area under the curve; GLCM = gray-level co-occurrence matrix; ICD = implantable cardioverter-defibrillator; LGE = late gadolinium enhancement.

## Materials and methods

### Study population

This is a retrospective study that consists of 270 patients with DCM (61% male, age 58 ± 13 years) from Cleveland Clinical Foundation (CCF), Cleveland, OH, for model development, and 113 patients with DCM (71% male, 55 ± 14 years) from Beth Israel Deaconess Medical Center (BIDMC), Boston, MA, for model validation. All patients were deemed eligible for primary ICD implantation by their treating cardiologists and underwent clinical CMR before primary prevention ICD implantation between 2008 and 2021. DCM diagnosis was established by their treating cardiologists based on medical history, physical examination, electrocardiography, and echocardiography. The cohort included 258 (96%) symptomatic individuals (NYHA functional class II-IV) from CCF and 98 (87%) from BIDMC. All patients exhibited either LV dilation or biventricular dilation with reduced LVEF, either at the time of the scan or at initial diagnosis in the clinic. Exclusion criteria included patients with ICM (history of myocardial infarction or prior coronary artery bypass surgery or ischemic LGE), mixed cardiomyopathy (significant coronary artery disease without a history of myocardial infarction), and the following nonischemic disease with a known diagnosis of specific high-risk syndromes: 1) hypertrophic cardiomyopathy; 2) arrhythmogenic right ventricular (RV) cardiomyopathy; 3) amyloidosis; 4) sarcoidosis; 5) Brugada syndrome; and 6) secondary prevention ICD recipients. The study was approved by the CCF and BIDMC Institutional Review Boards, and written consent was waived. All anonymized Digital Imaging and Communications in Medicine images were transferred to BIDMC for model development and evaluation.

### Cardiovascular magnetic resonance imaging

All CCF CMR images were acquired with a 1.5-T or 3-T scanner (Achieva 1.5-T, Ingenia 3-T, Philips Healthcare). BIDMC CMR images were acquired with 1.5-T (Achieva) or 3-T scanner (Magnetom Vida 3-T, Siemens Healthineers). The imaging protocol included a balanced steady-state free-precession cine sequence and LGE. LGE images were acquired 10 to 20 minutes after injection of 0.1 to 0.2 mmol/kg of gadolinium-based contrast agents (Magnevist, MultiHance, Gadoterate Meglumine). Protocol details are available in the Supplemental Material.

One reader from each site analyzed CMR images blinded to ICD therapy using cvi42 (version 5.14.1, Circle Cardiovascular Imaging Inc). The LV and RV endocardial and LV epicardial borders were manually traced on short-axis cine images to calculate LV and RV end-diastolic volume, end-systolic volume, ejection fraction, and mass. LGE images were assessed qualitatively and quantitatively. For quantitative assessment, a region of interest (ROI) was placed in an apparently normal area of the short-axis image to define normal myocardium. An automated computer-aided threshold detection at 5 SDs above the mean signal intensity of normal myocardium was used to identify LGE volume.

### Clinical follow-up and primary outcome

The primary endpoint of this study was the delivery of appropriate ICD therapy, including ICD shock or antitachycardia pacing for fast VT. Appropriate events were deﬁned as interventions for accurately detecting ventricular fatal arrhythmias. An electrophysiologist in each site blinded to CMR findings conducted adjudication of stored ICD electrograms. Device interrogation occurred 1 and 3 months postimplantation, followed by assessments every 6 months in the Device Clinic. The first evaluation date was the time of the CMR examination. The mean follow-up duration from study enrollment to the latest assessment (clinic visit, phone interview, or death) was 4.4 years in CCF and 2.9 years in BIDMC. Detailed prognostic data (vital status, clinical, and survival) were collected up to December 20, 2022, through hospital visits or communication with patients, family, and referring physicians.

### Region of interest segmentation and radiomic features

**Figure 1:**
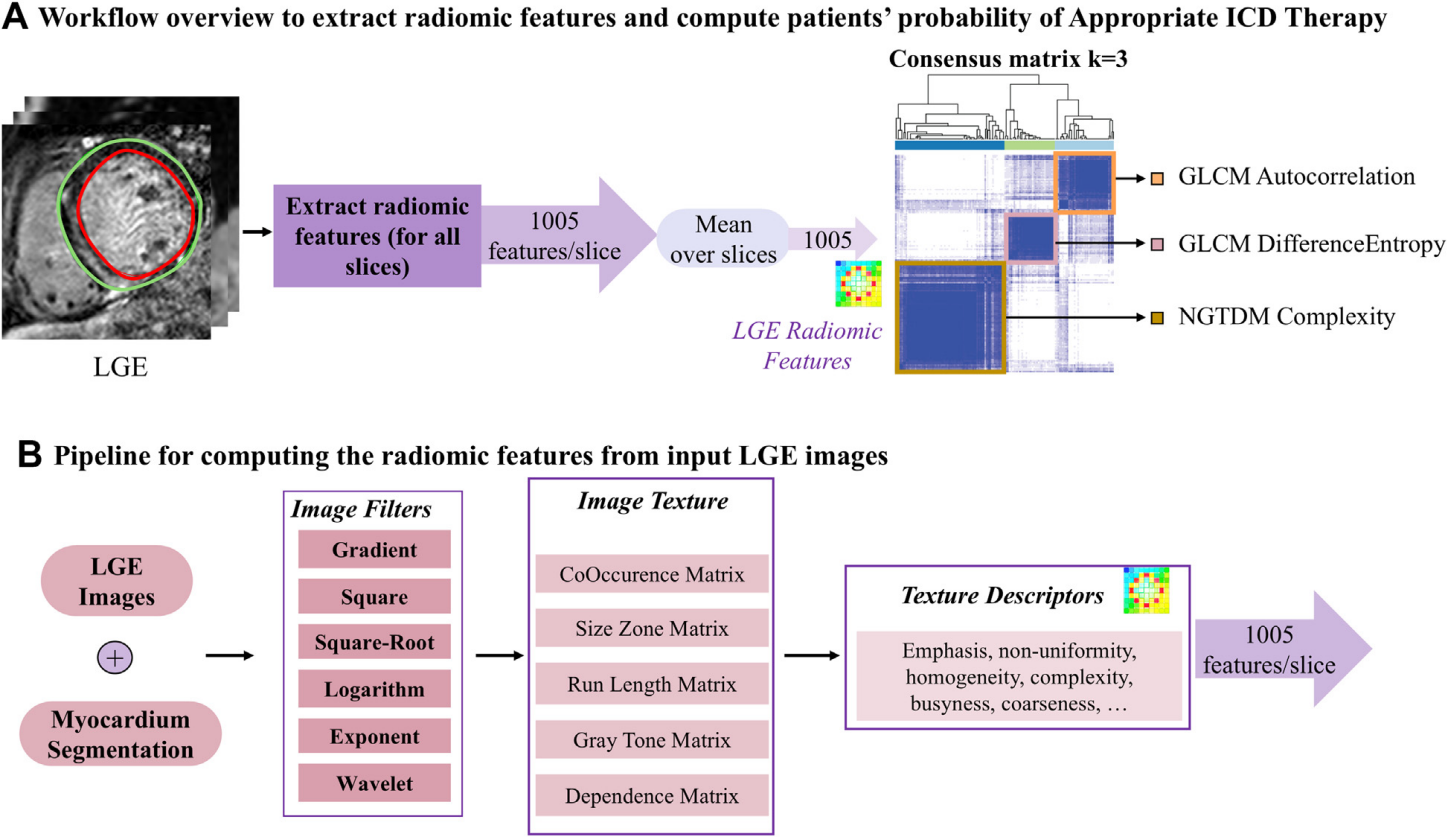
Radiomic Workflow for Feature Extraction and Patient Stratification The analytical framework employed for extracting late gadolinium enhancement radiomics (A). The sequence for computing radiomic features from input late gadolinium enhancement short-axis slices is outlined in (B). GLCM = gray level co-occurrence matrix; ICD = implantable cardioverter-defibrillator.

Defining an ROI and extracting pixel values are essential to computing radiomic features. We used the myocardium contours traced on short-axis LGE images as ROI (Figure 1A). Radiomic features were extracted from each slice containing the myocardium on LGE images, encompassing both healthy and scarred myocardium if present. This approach removed the need for LGE scar segmentation, offering a more thorough representation of the myocardium within the LGE images. We standardized the spatial resolution of the ROI to 1 × 1 mm^2^ through bilinear interpolation and normalized the intensity values within the ROI to a range of 0 to 1 using min-max values. Intensity normalization was necessary to standardize the dynamic range of all ROIs across all patients, irrespective of whether they were scanned using magnitude- or phase-sensitive inversion recovery LGE sequences. Features were extracted from the original ROI, along with 10 filtered versions (using exponential, gradient, logarithmic, squaring, square-root, local binary pattern, and 4 wavelet-transform filters), and 5 feature-family filters [gray level co-occurrence matrix (GLCM), Gray Level Size Zone Matrix, Gray Level Run Length Matrix, Neighboring Gray Tone Difference Matrix (NGTDM), gray level dependence matrix]. Radiomic features were computed using the open-source library Pyradiomics (version 3.0.1).^18^ We calculated the mean value across short-axis slices for each patient, resulting in 1,005 radiomic features characterizing the texture of each patient's LGE (Figure 1B). To address redundancy in the radiomic features, we eliminated highly correlated features with a correlation coefficient exceeding 0.8.^19^

### Consensus hierarchical clustering

We employed consensus hierarchical clustering^20^ to construct a radiomic signature that separated features based on their similarities. This methodology, being unsupervised and data-driven (ie, blinded to patient's outcome), aims to determine the ideal number of clusters (k) within a data set without relying on specific ground truth or outcomes. Instead of arbitrarily selecting k, the method involves iteratively subsampling 80% of the data set and performing hierarchical clustering on each subsample. The consensus matrix evaluates the relative frequency of feature clustering for each k value. To determine the optimal k value, indicative of a point where further improvements in separability become marginal, we computed the change in the area under the cumulative distribution function curve of the consensus matrix distribution for each k value. Consensus clustering was performed using the R package ConsensusClusterPlus.^21^ We then identified a single representative feature for each cluster by computing the medoid, representing the feature with the highest average pairwise correlation within the cluster. The medoid was obtained by applying the following equation: $\underset{\;y\in X}{x_{medoid}=\arg\;\;\min}\sum_{i=1}^{n}d\left(y,xi\right)$, where *X* is a set of n radiomic features, and d is the Euclidean distance function.

### Clinical score

The clinical risk score^22^ was calculated by using demographic, rhythm status, general cardiac status, and laboratory variables as the following formula: Clinical risk score = −0.2 × age + 4 × male + 5 × atrial fibrillation − 8 × amiodarone + 7 × pre-existing pacemaker − 3 × smoking – 0.2 × |QRS duration – 130| − 8 × anemia (hemoglobin <12 g/dL) + 1.77 × creatinine. The clinical risk score introduced in^22^ also includes nonsustained VT and digoxin; however, these variables were missing from our data set and thus excluded.

### LGE radiomics risk prediction

To evaluate the incremental value of radiomics, we built several models: the LGE burden model, LGE presence model, and a combination of the clinical score with either LGE burden or LGE presence. We then combined radiomic features with the clinical score and LGE burden/presence to build new models to assess the additive value of radiomics. All model development was conducted using CCF data exclusively (Central Illustration). Finally, we evaluated the performance of these models using the external validation data set from BIDMC. Models' implementation is publicly available at https://github.com/HMS-CardiacMR/NICM_ICD_Radiomics.

### Statistical analysis

Continuous variables are expressed as mean ± SD and compared using either an unpaired Student *t*-test or Mann-Whitney nonparametric test if not normally distributed. Categorical variables were reported as counts and percentages and compared using either a chi-squared test or Fisher exact test, as appropriate. The C-statistic or area under the roc curve was calculated for all models to predict appropriate ICD therapy; C-statistics for all models in the development cohort were corrected using bootstrapping of 1,000 samples. The optimal cutoff point of the model with the highest C-statistic was determined using the Youden index on the development cohort. We used the cutoff threshold to categorize the model predictions for each patient as low or high risk of appropriate ICD therapy. Reclassification of patients was determined through net reclassification improvement (NRI) analysis for appropriate ICD therapy, achieved by integrating radiomics into the clinical score and LGE models. Univariable and multivariable Cox proportional hazards analyses were used to evaluate the association between radiomic features, clinical score and LGE presence/burden, and the endpoint appropriate ICD therapy. The proportional hazards assumption was tested in the Cox model using Schoenfeld residuals, with a significance level of *P* = 0.05. All reported associations in this study are HRs and their corresponding 95% CIs. Kaplan-Meier curves were plotted to compare arrhythmia-free survival between high and low-risk appropriate ICD therapy groups. Time to event was calculated starting from the index CMR visit, and differences between time-to-event curves were assessed via the log-rank test. All tests were 2 sided and *P* value < 0.05 was considered significant. The DeLong test was used to compare areas under the receiver-operating characteristic curve. All data analysis was performed using Python 3.9.6. Statistical analyses were performed using the Python libraries scikit-learn (version 0.19.1), scipy-stats (version 1.12.0) and lifelines (version 0.28.0), and R (version 4.2.2).

## Results

### Baseline characteristics of study patients

The clinical and demographic characteristics of the study cohorts are summarized in Table 1. Appropriate ICD therapy occurred in 40 (15%) in development and 16 (15%) in external validation cohorts over a median follow-up period of 4.0 (IQR: 2.5-6.1) and 2.6 (IQR: 1.2-4.1) years, respectively. CMR findings between the 2 groups are detailed in Table 2. In the development cohort, LVEF was 25% ± 8%, with LGE present in 56% of patients, occupying on average 4.5% of LV mass. In the external cohort, the mean LVEF was 29% ± 12%, with LGE identified in 56% of patients and covering 3.6% of LV mass. LGE was also more prevalent in patients with appropriate ICD therapy (development cohort: *P* = 0.004, validation cohort: *P* = 0.04). Moreover, patients with appropriate ICD therapy showed a trend toward lower right ventricular ejection fraction (RVEF) (development, *P* = 0.02; validation cohort, *P* = 0.22), with no significant differences in LV function and morphology (all *P* > 0.05).

**Table 1:** Patient Characteristics Grouped by Appropriate Implantable Cardioverter Defibrillator Therapy

	Development Cohort				External Validation Cohort			
	All Patients (n = 270)	Patients With Event (n = 40, 15%)	Patients Without Event (n = 230)	*P* Value	All Patients (n = 113)	Patients With Event (n = 16, 15%)	Patients Without Event (n = 97)	*P* Value
Demographics								
Age, y	58 ± 13	55 ± 14	58 ± 13	0.10	55 ± 14	51 ± 16	55 ± 13	0.21
Male	164 (61)	28 (70)	136 (59)	0.93	80 (71)	14 (88)	66 (68)	0.98
Body surface area, m^2^	2.01 ± 0.3	2.04 ± 0.3	2.01 ± 0.3	0.50	2.03 ± 0.3	2.06 ± 0.3	2.02 ± 0.3	0.63
Body mass index, kg/m^2^	29.3 ± 6.0	28.7 ± 5.8	29.3 ± 6.1	0.55	28.6 ± 5.8	28.1 ± 7.1	28.7 ± 5.6	0.68
Hypertension	145 (54)	21 (53)	124 (54)	0.50	57 (50)	7 (44)	50 (52)	0.38
Diabetes mellitus	50 (19)	6 (15)	46 (20)	0.31	21 (19)	2 (13)	19 (20)	0.39
History of stroke	24 (9)	2 (5)	22 (10)	0.28	5 (4)	1 (6)	4 (4)	0.85
Atrial fibrillation	41 (15)	8 (20)	33 (14)	0.87	21 (19)	6 (38)	15 (15)	0.07
NYHA functional class				0.21				0.20
II	173 (64)	24 (60)	149 (65)		80 (71)	12 (75)	68 (70)	
III	85 (32)	14 (35)	71 (31)		18 (16)	3 (19)	15 (15)	
Time between CMR and implantation, d	93 ± 86	73 ± 71	96 ± 88	0.06	128 ± 140	116 ± 149	130 ± 139	0.37
Sodium, mmol/L	139 ± 3	139 ± 3	139 ± 3	0.54	140 ± 3	137 ± 3	140 ± 3	0.01
Serum BUN, mg/dL	20 ± 9	21 ± 9	20 ± 9	0.73	20 ± 8	19 ± 6	20 ± 8	0.33
Serum creatinine, mg/dL	1.1 ± 0.3	1.1 ± 0.3	1.1 ± 0.3	0.82	1.0 ± 0.2	1.0 ± 0.2	1.0 ± 0.2	0.35
Hemoglobin, g/dL	13.5 ± 1.6	13.7 ± 1.7	13.5 ± 1.5	0.51	14.0 ± 2.7	14.2 ± 1.4	14.0 ± 2.9	0.29
NT-proBNP, pmol/L	1,303 (47-14,889)	957 (82-3,318)	1,357 (47-14,889)	0.28	2,061 (41-12,743)	2,397 (156-9,205)	1,998 (41-12,743)	0.21
ECG parameter								
QRS duration, ms	130 ± 35	122 ± 30	131 ± 35	0.08	119 ± 28	117 ± 23	120 ± 29	0.45
Medication use								
ACEI, ARB, ARNI	250 (93)	39 (98)	211 (92)	0.96	92 (81)	12 (75)	80 (82)	0.34
Beta-blocker	257 (95)	39 (98)	218 (95)	0.88	99 (88)	12 (75)	87 (90)	0.11
Amiodarone	14 (5)	5 (13)	9 (4)	0.89	11 (9)	3 (19)	8 (8)	0.95
Statin	116 (43)	15 (38)	101 (44)	0.28	45 (40)	5 (31)	40 (41)	0.32

Values are mean ± SD, n (%), or median (IQR).

ACE = angiotensin-converting enzyme; ARB = angiotensin receptor blockers; ARNIs = angiotensin receptor-neprilysin inhibitors; BUN = blood urea nitrogen; CMR = cardiovascular magnetic resonance; ECG = electrocardiogram; NT-proBNP = N-terminal pro-B-type natriuretic peptide.

**Table 2:** CMR Findings Grouped by Appropriate Implantable Cardioverter Defibrillator Therapy

	Development Cohort				External Validation Cohort			
	All Patients (N = 270)	Patients With Event (n = 40, 15%)	Patients Without Event (n = 230)	*P* Value	All Patients (N = 113)	Patients With Event (n = 16, 15%)	Patients Without Event (n = 97)	*P* Value
LV EDV, ml	308 ± 103	312 ± 84	307 ± 106	0.25	285 ± 107	307 ± 132	282 ± 102	0.36
LV EDVI, mL/m^2^	153 ± 48	152 ± 36	153 ± 50	0.36	140 ± 45	149 ± 56	138 ± 43	0.33
LV ESV, ml	234 ± 94	240 ± 77	233 ± 97	0.19	209 ± 104	230 ± 133	206 ± 99	0.34
LV EF, %	25 ± 8	24 ± 7	26 ± 9	0.15	29 ± 12	29 ± 13	30 ± 12	0.36
LV EF ≤35%	248 (92)	38 (95)	210 (91)	0.87	85 (75)	11 (69)	74 (76)	0.36
LV EF ≤30%	186 (69)	30 (75)	156 (68)	0.86	69 (61)	10 (63)	59 (61)	0.65
LV mass, g	164 ± 49	168 ± 44	164 ± 50	0.25	160 ± 57	160 ± 64	159 ± 57	0.48
LV mass index, g/m^2^	82 ± 23	82 ± 17	81 ± 24	0.22	78 ± 26	77 ± 28	79 ± 26	0.30
LV LGE	151 (56)	31 (78)	126 (55)	0.004	63 (56)	12 (75)	51 (53)	0.04
LV LGE burden, %	4.5 ± 5.7	5.7 ± 6.3	4.3 ± 5.6	0.01	3.6 ± 5.8	3.6 ± 3.9	3.6 ± 6.1	0.11
RV EDV, ml	184 ± 66	192 ± 60	183 ± 67	0.09	177 ± 66	210 ± 88	172 ± 61	0.08
RV ESV, ml	117 ± 61	126 ± 55	116 ± 62	0.15	105 ± 63	136 ± 90	99 ± 57	0.16
RVEF, %	39 ± 13	37 ± 12	40 ± 13	0.02	45 ± 14	41 ± 17	45 ± 13	0.22

Values are mean ± SD or n (%).

EDV = end-diastolic volume; EDVI = end-diastolic volume index; EF = ejection fraction; ESV = end-systolic volume; LGE = late gadolinium enhancement; LV = left ventricular; RV = right ventricular.

### Consensus hierarchical clustering radiomics

**Figure 2:**
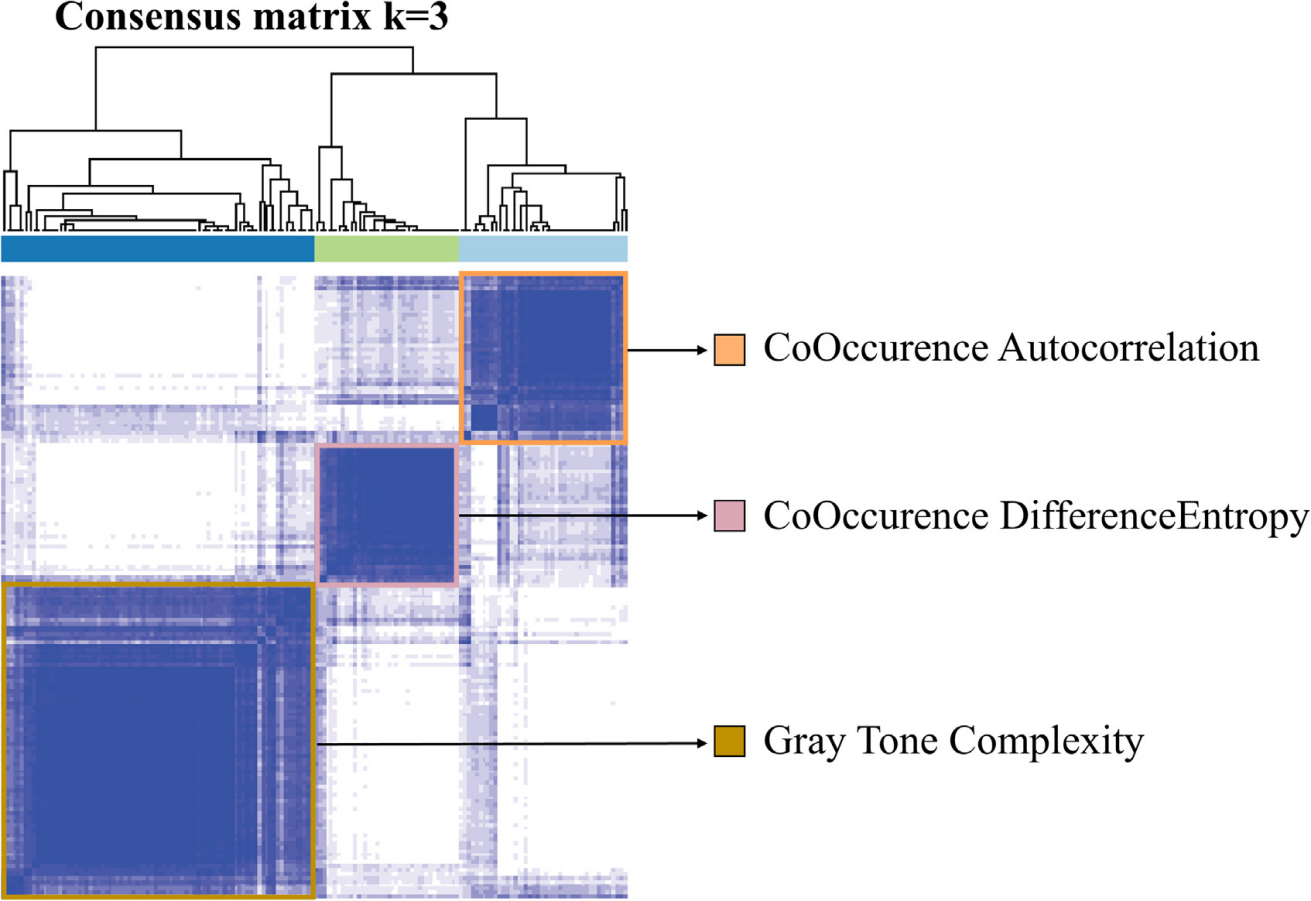
Consensus Clustering of Radiomic Features Using a Conditional Density Function at a k Value of 3 To determine the optimal number of clusters (k), an iterative subsampling of 80% of the data set was performed and a hierarchical clustering to each subsample was applied. For each k value within a range of 1 to 6, the relative frequency of patients clustering relative to others was evaluated, resulting in the creation of a consensus matrix. Subsequently, to obtain the degree of cluster separation, the change in the area under the cumulative distribution function curve of the consensus matrix for each k value was calculated. The optimal k value was then determined based on a significant decrease in this change, as indicated by the area under the receiver operating characteristic curve. This finding suggested that further improvements in cluster separability beyond the selected k value yield only marginal improvements.

Consensus hierarchical clustering analysis demonstrated a clear reduction in the relative change within the area under the conditional density function curve at a k value of 3 (Figure 2). In cluster 1, the representative radiomic feature was the autocorrelation derived from the GLCM. For cluster 2, the representative feature was the difference entropy from the GLCM, and for cluster 3, the representative feature was complexity from the NGTDM. The radiomic signature was composed by combining these 3 distinct features.

### Clinical and LGE burden risk prediction model

In the development cohort, the C-statistics for the clinical and LGE burden model was 0.61 (95% CI: 0.53-0.71) (Table 3). In the external validation cohort, the C-statistic for the model was 0.62 (95% CI: 0.46-0.77). Notably, the integration of radiomic features with clinical and scar burden significantly improved performance (C-statistics) to 0.71 (95% CI: 0.62-0.80) for development and 0.68 (95% CI: 0.57-0.83) for external validation (*P* = 0.045, *P* = 0.03, respectively). This also significantly improved risk stratification with a continuous NRI of 0.60 (95% CI: 0.28-0.92; *P* < 0.001) in development and of 0.20 (95% CI: 0.17-0.34; *P* = 0.04) in the validation cohort. In particular, the addition of radiomics yielded 3 (33%) correct (up) and 3 (10%) incorrect (down) reclassification in the 40 patients with appropriate ICD events in development. There were 34 (27%) correct (down) and 20 (19%) incorrect (up) reclassifications that occurred in the 230 patients who did not have events. In the validation cohort, the addition of radiomics yielded 6 (46%) correct (up) and 1 (33%) incorrect (down) reclassification in the 16 patients with appropriate ICD events in development. There were 7 (47%) correct (down) and 24 (29%) incorrect (up) reclassification that occurred in the 97 patients who did not have events.

**Table 3:** Performance Evaluation of Risk Stratification Models for Appropriate Implantable Cardioverter Defibrillator Therapy With and Without Integrating Radiomics Features in the Development and External Validation Cohorts

	Development Cohort		External Validation Cohort	
	Without Radiomics	With Radiomics	Without Radiomics	With Radiomics
Radiomics-only	-	0.68 (0.65-0.70)	-	0.70 (0.54-0.85)
LGE presence	0.61 (0.55-0.70)	0.70^a^ (0.67-0.71)	0.61 (0.47-0.75)	0.69 (0.53-0.84)
LGE burden	0.59 (0.39-0.61)	0.68^a^ (0.64-0.70)	0.59 (0.45-0.73)	0.66 (0.50-0.81)
Clinical + LGE burden	0.59 (0.49-0.61)	0.68^a^ (0.63-0.70)	0.62 (0.46-0.77)	0.68^a^ (0.57-0.83)
Clinical + LGE presence	0.61 (0.58-0.61)	0.70^a^ (0.66-0.71)	0.61 (0.46-0.77)	0.70^a^ (0.59-0.85)

Data represent the area under the receiver-operating characteristic curve (95% CI) of the receiver-operating characteristics.

AUC = area under the curve; other abbreviations as in Table 2.

a Statistical significance by DeLong's test of comparing the AUCs of the clinical features and LGE models *with* vs *without* integrating radiomics features.

### Clinical and LGE presence risk prediction model

**Figure 3:**
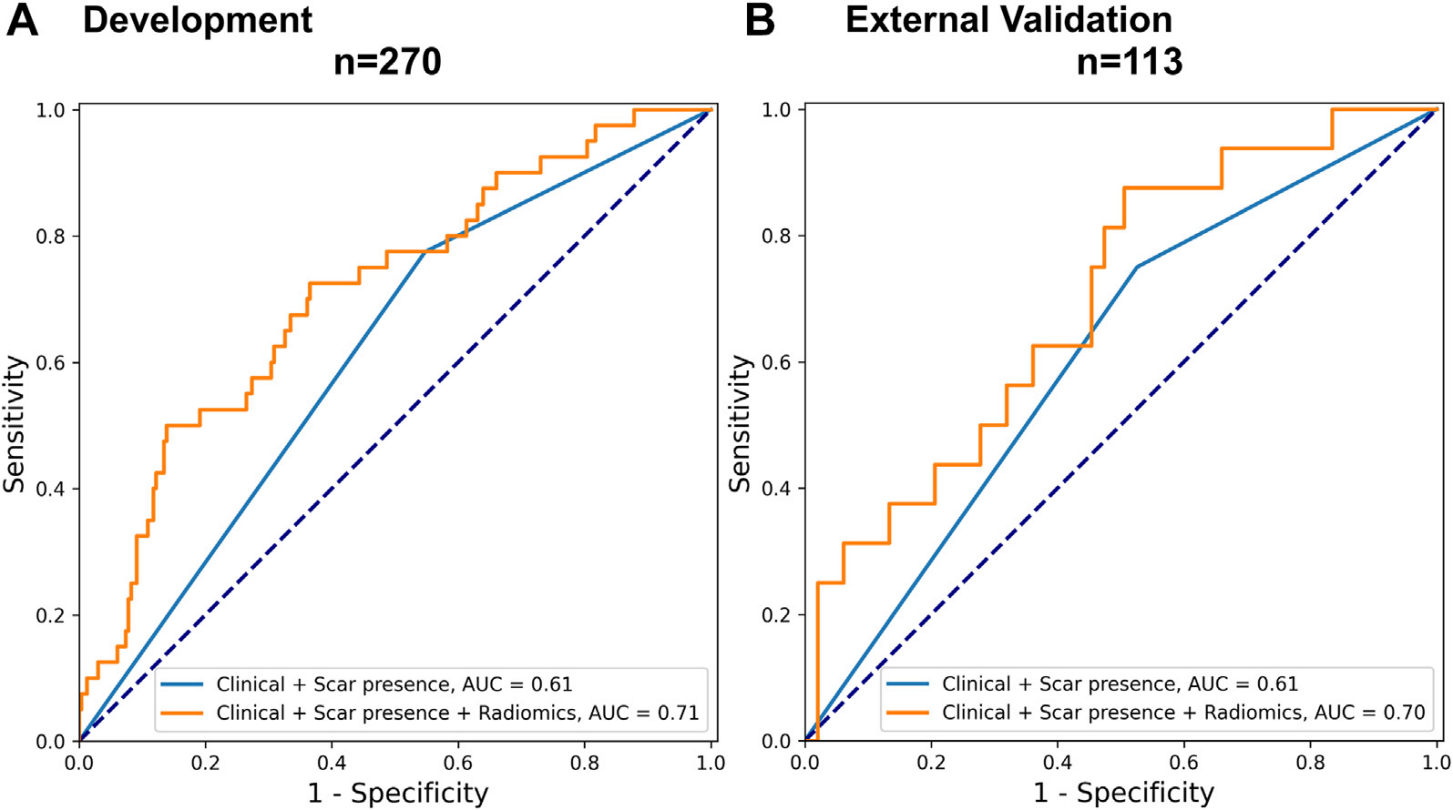
Receiver-Operating Characteristics Curves for the Risk Prediction Models of Appropriate Implanted Cardioverter Defibrillator Therapy, Integrating Radiomics With Clinical Assessment and Scar Presence (A) Development data set and (B) external validation data set. AUC = area under the curve.

Similarly, the combination of clinical and LGE presence model had a C-statistic of 0.61 (95% CI: 0.53-0.71) and 0.61 (95% CI: 0.46-0.77) in the development and external validation cohort, respectively. This increased to 0.71 (95% CI: 0.62-0.80) and 0.70 (95% CI: 0.59-0.85) with the addition of radiomics (development: *P* = 0.02; external validation: *P* = 0.003) (Figure 3). It also significantly improved risk stratification with a continuous NRI of 0.60 (95% CI: 0.29-0.91; *P* < 0.001) in development and of 0.29 (95% CI: 0.26-0.56; *P* = 0.03) in external validation. In particular, the addition of radiomics yielded 17 (63%) correct (up) and 3 (23%) incorrect (down) reclassification in the 40 patients with appropriate ICD events in development. There were 30 (50%) correct (down) and 53 (31%) incorrect (up) reclassifications that occurred in the 230 patients who did not have events. In the validation cohort, the addition of radiomics yielded 3 (75%) correct (up) and 1 (8%) incorrect (down) reclassification in the 16 patients with appropriate ICD events in development. There were 23 (45%) correct (down) and 20 (43%) incorrect (up) reclassifications that occurred in the 97 patients who did not have events.

### Survival analysis and impact of radiomic features

Univariate and multivariate Cox proportional hazard analyses for appropriate ICD therapy are summarized in Tables 4 and 5. The 3 radiomic features were associated with appropriate ICD therapy in the development cohort (HR: 1.53; 95% CI: 1.17-2.00; *P* < 0.005, HR: 1.71; 95% CI: 1.18-2.48; *P* < 0.005, and HR: 1.62; 95% CI: 1.10-2.41; *P* = 0.02, respectively). Patients with LGE had a 191% higher risk of an appropriate ICD therapy (HR: 2.91; 95% CI: 1.39-6.12; <0.005). In the external validation cohort, only the first radiomic feature GLCM autocorrelation had a 164% higher risk of an appropriate ICD therapy (HR: 2.64; 95% CI: 1.47-4.75; *P* < 0.005). The presence of LGE was associated with a 158% higher risk of an appropriate ICD therapy (HR: 2.58; 95% CI: 0.83-8.03: *P* = 0.10).

**Table 4:** Cox Regression Analysis of the Radiomic Features, Clinical, and the Presence/Burden of LGE for Appropriate ICD Therapy in the Development Cohort

Model Predictor	Univariate		Clinical + LGE Presence +		Clinical + LGE Burden +	
	Model		Radiomics Model^a^		Radiomics Model^a^	
	HR (95% CI)	*P* Value	HR (95% CI)	*P* Value	Hazard Risk Ratio [95% CI]	*P* Value
GLCM autocorrelation	1.53 (1.17-2.00)	<0.005	1.45 (1.10-1.91)	0.01	1.43 (1.09-1.86)	0.01
GLCM difference entropy	1.71 (1.18-2.48)	<0.005	1.34 (0.88-2.02)	0.17	1.55 (1.05-2.29)	0.03
NGTDM complexity	1.62 (1.10-2.41)	0.02	1.47 (0.97-2.24)	0.07	1.50 (1.01-2.25)	0.05
LGE presence	2.91 (1.39-6.12)	<0.005	2.55 (1.19-5.47)	0.02	-	-
LGE burden	1.05 (1.00-1.10)	0.03	-	-	1.06 (1.01-1.11)	0.01

Variables are hazard ratios with 95% confidence intervals.

GLCM = gray level co-occurrence matrix; other abbreviations as in Table 2.

a Radiomics model: combination of GLCM autocorrelation, GLCM difference entropy, and NGTDM complexity.

**Table 5:** Cox Regression Analysis of the Radiomic Features, Clinical, and the Presence/Burden of LGE for Appropriate ICD Therapy in the External Validation Cohort

Model Predictor	Univariate		Clinical + LGE Presence +		Clinical + LGE Burden +	
	Model		Radiomics Model^a^		Radiomics Model^a^	
	Hazard Risk Ratio (95% CI)	*P* Value	Hazard Risk Ratio (95% CI)	*P* Value	Hazard Risk Ratio (95% CI)	*P* Value
GLCM Autocorrelation	2.64 (1.47-4.75)	<0.005	2.38 (1.28-4.42)	0.01	2.52 (1.35-4.71)	<0.005
GLCM Difference Entropy	1.59 (0.91-2.78)	0.10	1.46 (0.74-2.86)	0.27	1.48 (0.75-2.93)	0.26
NGTDM Complexity	1.13 (0.69-1.86)	0.63	0.97 (0.55-1.70)	0.91	0.88 (0.0-1.53)	0.64
LGE presence	2.58 (0.83-8.03)	0.10	2.16 (0.66-7.10)	0.21	-	-
LGE burden	1.00 (0.92-1.09)	0.92	-	-	1.00 (0.91-1.10)	0.97

Variables are hazard ratios with 95% confidence intervals.

Abbreviations as in Table 4.

a Radiomics model: combination of GLCM autocorrelation, GLCM difference entropy, and NGTDM complexity.

**Figure 4:**
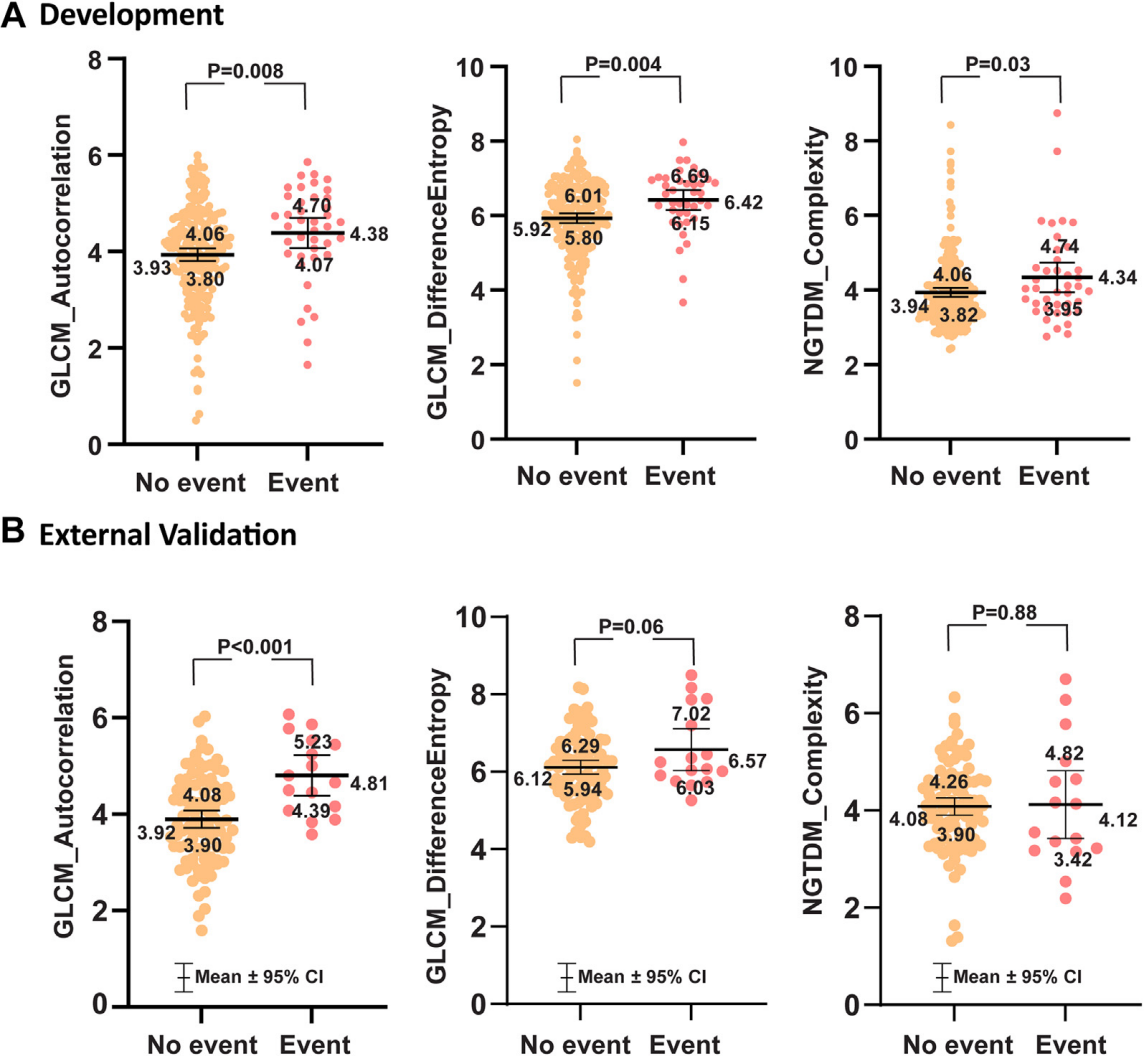
Scatter Plot of Radiomic Features Comparing Patients With and Without Events Scatter plot with mean ± 95% CI for (A) development data and (B) external validation data for the 3 radiomic features analyzed in the study. The mean and distribution of the gray level co-occurrence matrix autocorrelation between patients with events (appropriate implanted cardioverter defibrillator therapy) and those without exhibit a statistically significant differentiation between the 2 groups. GLCM = gray level co-occurrence matrix.

**Figure 5:**
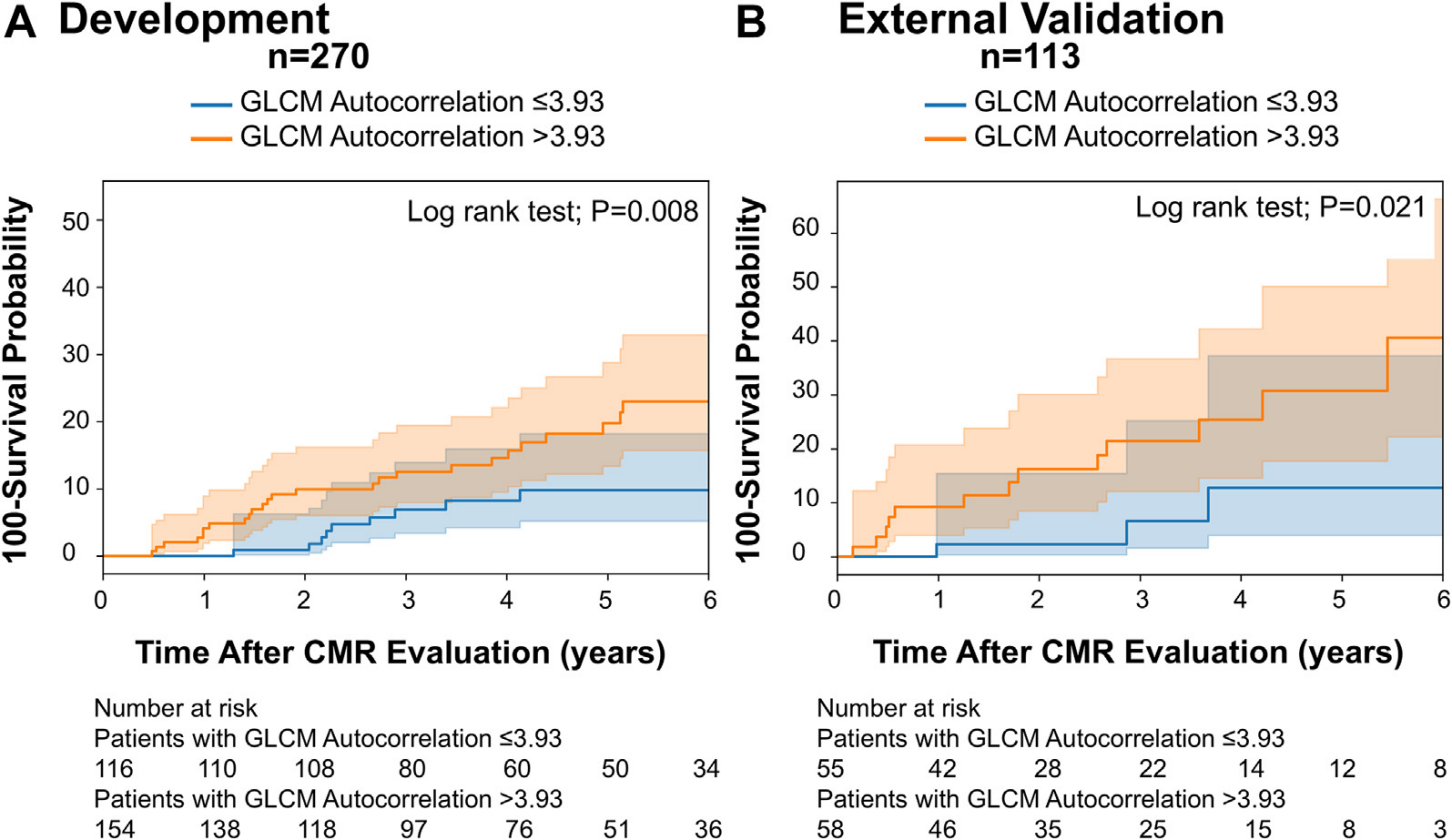
Time to-Event Analysis for Appropriate Implanted Cardioverter Defibrillator Therapy Time to-event analysis for appropriate implanted cardioverter defibrillator therapy in (A) development data set and (B) external validation data set shows an excellent separation Between high-risk and low-risk groups at a gray level co-occurrence matrix autocorrelation threshold of ≤3.93. CMR = cardiovascular magnetic resonance; GLCM = gray level co-occurrence matrix.

After adjusting for clinical score and LGE, only the GLCM autocorrelation remained significant among the radiomic features with a 45% risk increase in development (HR: 1.45; 95% CI: 1.10-1.91; *P* = 0.01), and an 138% risk increase in external validation (HR: 2.83; 95% CI: 1.28-4.42; *P* = 0.01). The mean value of the GLCM autocorrelation in the appropriate ICD therapy group was significantly higher than the group without event in development cohort 4.38 (95% CI: 4.07-4.70) vs 3.93 (95% CI: 3.80-4.06) (*P* = 0.008), and in external validation 4.81 (95% CI: 4.39-5.23) vs 3.92 (95% CI: 3.90-4.08) (*P* < 0.001) (Figure 4). The optimal GLCM autocorrelation threshold separating the high-risk appropriate ICD therapy group from low-risk using the Youden index on the development cohort was 3.93. Kaplan-Meier curves illustrated excellent arrhythmia-free survival in patients with GLCM autocorrelation ≤3.93 in both development and external validation cohorts (Figure 5).

## Discussion

In this 2-center multivendor study of 383 patients with DCM who received an ICD for primary prevention under current guidelines, we demonstrated that: 1) only a minority of patients experienced appropriate ICD therapy, highlighting the limitations of current guidelines and the increased morbidity for patients who do not benefit; and 2) radiomic analysis of LGE adds significant incremental value beyond clinical and LGE assessment. Importantly, we demonstrated the generalizability of these findings on an external cohort of patients from a different site and vendor. By identifying patients with a low radiomic risk value, we may be able to prevent unnecessary morbidity for those who would not benefit from ICD and reduce societal costs.

Despite the effectiveness and established status of ICDs in treating life-threatening ventricular arrhythmias, the current guidelines result in only a minority of primary prevention ICD recipients receiving appropriate therapy.^2^, ^3^, ^4^, ^5^ This shortfall contributes to elevated societal costs and increased patient morbidity. There are several challenges associated with predicting VT/VF in patients with DCM. The progression of DCM can vary widely among individuals, with some patients experiencing rapid deterioration while others remain relatively stable.^23^ Predicting VT/VF risk requires accounting for these variable disease trajectories and identifying patients at higher risk of arrhythmias. Thus, incorporating individual patient characteristics and clinical variables into predictive models should improve risk stratification.^24^ However, a limited performance was observed when using the clinical score alone in predicting VT/VF.^25^ Moreover, despite the high risk associated with LGE, its presence and burden had limited predictive power in discriminating between high and low-risk groups. LGE is present in 40% of patients with DCM with a distinct mid-myocardial presence.^26^^,^^27^ Nevertheless, arrhythmias could occur in patients without any evidence of LGE, and little is known about the substrate of arrhythmia in patients with DCM with and without LGE. The findings presented in our study have the potential to address this challenge by introducing an improved risk stratification model. Indeed, radiomics analysis of the homogeneity and heterogeneity of the myocardium on LGE provided a significantly improved risk stratification model.

Radiomic analysis typically entails extracting thousands of features from an ROI, resulting in a high-dimensional feature space. Given the high correlation among many radiomic features, it is advisable to exclude strongly correlated ones to prevent redundancy.^19^ Subsequently, a supervised feature selection technique is typically employed to identify the most informative features, forming a radiomic signature. Although this approach is powerful in learning the relationship between features and outcomes, it is prone to overfitting and leakage of outcome information during training. Unsupervised feature selection techniques offer a potential solution to this issue by operating solely at the feature level without prior information about the outcome.^16^^,^^28^ We used consensus clustering, which involves grouping extracted radiomic features based on their similarities without previous knowledge of the outcome. By calculating pairwise dissimilarities within each group, we extracted a single representative feature per group to form the radiomic signature. This methodology aimed to minimize subjective influences and enhance objectivity, resulting in more robust and unbiased outcomes.^16^^,^^29^ Acknowledging the critical importance of the generalizability and reproducibility of our findings, we sought to independently validate the model in an external cohort of 113 patients from a different center, with images obtained by a different scanner.

Among the representative radiomic features identified in our study, the GLCM autocorrelation demonstrated significant prognostic value in both the development and validation cohorts. GLCM-based features have been reported for their high reproducibility in T_1_/T_2_ weighted images and T_1_/T_2_ maps.^30^ Fahmy et al^16^ showed that the NGTDM-based feature, coarseness, has significant prognostic value in predicting sudden cardiac death in patients with hypertrophic cardiomyopathy. GLCM quantifies the spatial relationship between pixel intensities, allowing for the analysis of gray level co-occurrence at different distances and angles within the image. This enables GLCM to capture variations and patterns representing heterogeneity, such as differences in texture, roughness, or composition. The autocorrelation feature from GLCM measures the texture's smoothness and roughness. A rough texture is characterized by large, prominent features with a grainy appearance. Our research indicates that a rough texture observed in myocardial LGE could be an indicator of a future appropriate ICD therapy events, contrasting with a smooth texture associated with a lack of ICD therapy. Although radiomics has garnered increased interest in CMR imaging and other fields, its translation to clinical practice is constrained by various factors, including interpretation of the features.^31^ GLCM autocorrelation, easily measured from LGE images, presents a single value that could be seamlessly integrated with other risk biomarkers. In our study, we established a cutoff for GLCM autocorrelation using a development cohort and subsequently validated this cutoff on an external validation cohort. Both cohorts exhibited excellent arrhythmia-free survival despite including 3T Siemens images in the validation cohort, a vendor different from development. Further studies are needed to validate our findings in a large and diverse cohort of patients using a variety of CMR vendors and field strengths.

The study's primary endpoint was the delivery of appropriate ICD therapy, including ICD shock or antitachycardia pacing for fast VT/VF, irrespective of the type of arrhythmia. Notably, the radiomic signature of the LGE was determined blinded to the outcome. All patients in our cohort met the criteria for primary prevention ICD implantation, primarily based on LVEF. Therefore, our model did not include LVEF as a risk marker. Recent studies have demonstrated a link between RV function and adverse outcomes in various cardiovascular diseases.^32^^,^^33^ Particularly, RVEF has been identified as an independent predictor of arrhythmic events in patients with mild to moderate LV dysfunction^34^ and as a predictor of all-cause mortality in the DANISH trial.^35^ More recently, Jimenez-Juan et al^36^ highlighted RVEF role in predicting appropriate shocks and mortality in ICD recipients, while Fahmy et al^37^ showed that RVEF, along with RV end-systolic and end-diastolic volumes (indicators of RV dysfunction and remodeling), are significant risk markers for cardiovascular hospitalization and all-cause mortality in patients with non-ICM. Further research is warranted to investigate the role of RV-based measurements in predicting appropriate ICD therapy for patients with DCM. Developing robust predictive models requires a thorough understanding of the intricate pathophysiology driving arrhythmogenesis in DCM, alongside validation across diverse patient cohorts. Alterations in conduction velocity, associated with scar formation and diffuse fibrosis, can lead to mechanical irregularities that are quantifiable through myocardial strain parameter assessment.^25^ Furthermore, research into genetics, proteomics, and blood biomarkers is ongoing to predict VT/VF in patients with DCM.^38^, ^39^, ^40^ Deep learning methods have also been explored to predict VT/VF in ICM.^41^ Further studies are warranted to examine the integration of LGE radiomics with these biomarkers and deep learning models to facilitate a comprehensive risk prediction assessment.

### Study limitations

Our study has several limitations. This retrospective study consists of patients who underwent imaging over an extended period, during which there were likely changes in standard ICD programming. However, most patients received their ICD with improved programming after 2012.^42^ ICD selection and programming were not standardized and were left to the operator's discretion. Our data set lacked information on nonsustained VT and digoxin usage, leading to their exclusion from the clinical score.

## Conclusions

This proof-of-concept study demonstrated that radiomics analysis of LGE, particularly a single feature quantifying the smoothness and roughness of texture, provides additional incremental value beyond clinical and LGE burden in arrhythmia risk stratification in DCM. Further larger multicenter prospective studies may be needed to confirm our preliminary findings.

## Funding support and author disclosures

Dr Nezafat has received grant funding from the 10.13039/100000002National Institutes of Health (NIH) 1R01HL129185, 1R01HL129157, 1R01HL127015, and 1R01HL154744 (Bethesda, MD, USA). Dr Amyar has received grant funding from the 10.13039/100000968American Heart Association (AHA) 24CDA1257995 (Waltham, MA, USA). Drs Nezafat and Manning have a research agreement with Siemens Healthineers, the manufacturer of MRI system used in a subset of patients for imaging. Dr Kwon has a research agreement with Circle, Cardiovascular Imaging. All other authors have reported that they have no relationships relevant to the contents of this paper to disclose.

Perspectives **COMPETENCY IN MEDICAL KNOWLEDGE:** In a 2-center multivendor cohort of patients with nonischemic dilated cardiomyopathy, radiomic analysis of myocardial late gadolinium enhancement can add an incremental prognostic value to current established ventricular tachycardia/ventricular fibrillation risk prediction models.

**TRANSLATIONAL OUTLOOK:** Large external data sets are needed to confirm the generalizability of our findings.
