# Supplementary material for: Radiomic Cardiac MRI Signatures for Predicting Ventricular Arrhythmias in Patients With Nonischemic Dilated Cardiomyopathy
Source: JACC Adv. 2025 Mar 23;4(4):101684. doi: 10.1016/j.jacadv.2025.101684 (PMC11980004; doi:10.1016/j.jacadv.2025.101684)
Supplement: Supplementary data [file mmc1.docx]

**SUPPLEMENTAL APPENDIX**

**Sensitivity, specificity, positive and negative predictive values and the positive and negative likelihood ratios of the clinical model and the clinical models with radiomic features**

The diagnostic performance of all models was evaluated using sensitivity (Sens), specificity (Spec), positive predictive value (PPV), negative predictive value (NPV), positive likelihood ratio (PLR), and negative likelihood ratio (NLR) in both the development and validation cohorts.

Table S1 shows results for both development and validation cohorts in predicting appropriate implantable cardioverter-defibrillator event.

**Development cohort.** The Clinical + LGE Presence model demonstrated a sensitivity of 0.75 and a specificity of 0.45, with a PPV of 0.20 and an NPV of 0.92 (PLR: 1.41; NLR: 0.50). The Clinical + LGE Burden model resulted in a lower sensitivity (0.65) but improved specificity (0.58), with a PPV of 0.21, NPV of 0.91, PLR of 1.56, and NLR of 0.60. The addition of radiomics to the Clinical + LGE Presence model improved specificity to 0.63 and maintained high sensitivity (0.73). This model also yielded an improved PPV of 0.25, an NPV of 0.93, a PLR of 1.96, and an NLR of 0.43. Similarly, the Clinical + LGE Burden + Radiomics model achieved a sensitivity of 0.65 and a specificity of 0.64, with a PPV of 0.24, NPV of 0.91, PLR of 1.82, and NLR of 0.54.

**Validation Cohort.** The Clinical + LGE Presence model showed a sensitivity of 0.69 and a specificity of 0.38, with a PPV of 0.15 and an NPV of 0.88 (PLR: 1.11; NLR: 0.82). The Clinical + LGE Burden model maintained a sensitivity of 0.69 but improved specificity to 0.53, with a PPV of 0.19, an NPV of 0.91, a PLR of 1.45, and an NLR of 0.59. Adding radiomics to the Clinical + LGE Presence model resulted in the highest diagnostic performance, with a sensitivity of 0.71, specificity of 0.64, PPV of 0.26, and NPV of 0.94 (PLR: 2.07; NLR: 0.39). The Clinical + LGE Burden + Radiomics model also achieved a sensitivity of 0.68, though specificity decreased to 0.59, with a PPV of 0.22, NPV of 0.92, PLR of 1.67, and NLR of 0.53.

**Decision Curve Analysis**

Decision curve analysis was performed to evaluate the clinical utility of the models in both the development and validation cohorts (**Figures S2, S3**). In the development cohort, models including radiomics consistently demonstrated higher net benefit compared to models without radiomics across a range of threshold probabilities. Specifically, the Clinical + LGE Burden + Radiomics and Clinical + LGE Presence + Radiomics models outperformed their counterparts without radiomics, particularly at lower to moderate threshold probabilities (<20%). This indicates that the inclusion of radiomics improves the model's ability to identify true positives while minimizing unnecessary interventions.

In the validation cohort, the radiomics-enhanced models continued to show higher net benefit compared to models without radiomics at lower threshold probabilities. Notably, the Clinical + LGE Presence + Radiomics model provided the greatest net benefit among all tested. Both radiomics-enhanced models outperformed the "treat-all" and "treat-none", demonstrating their added value in guiding clinical decision-making.

**Supplemental Table 1.** **Patient characteristics grouped by appropriate** **ICD therapy.**

|  | Sens | Spec | PPV | NPV | PLR | NLR |
| --- | --- | --- | --- | --- | --- | --- |
| Development cohort | | | | | | |
| Clinical + LGE presence | 0.75 | 0.45 | 0.20 | 0.92 | 1.41 | 0.50 |
| Clinical + LGE burden | 0.65 | 0.58 | 0.21 | 0.91 | 1.56 | 0.60 |
| Clinical + LGE presence + Radiomics | 0.73 | 0.63 | 0.25 | 0.93 | 1.96 | 0.43 |
| Clinical + LGE burden + Radiomics | 0.65 | 0.64 | 0.24 | 0.91 | 1.82 | 0.54 |
| Validation cohort | | | | | | |
| Clinical +LGE presence | 0.69 | 0.38 | 0.15 | 0.88 | 1.11 | 0.82 |
| Clinical +LGE burden | 0.69 | 0.53 | 0.19 | 0.91 | 1.45 | 0.59 |
| Clinical + LGE presence + Radiomics | 0.71 | 0.64 | 0.26 | 0.94 | 2.07 | 0.39 |
| Clinical + LGE burden + Radiomics | 0.68 | 0.59 | 0.22 | 0.92 | 1.67 | 0.53 |

sensitivity (Sens), specificity (Spec), positive predictive value (PPV), negative predictive value (NPV), positive likelihood ratio (PLR), negative likelihood ratio (NLR)


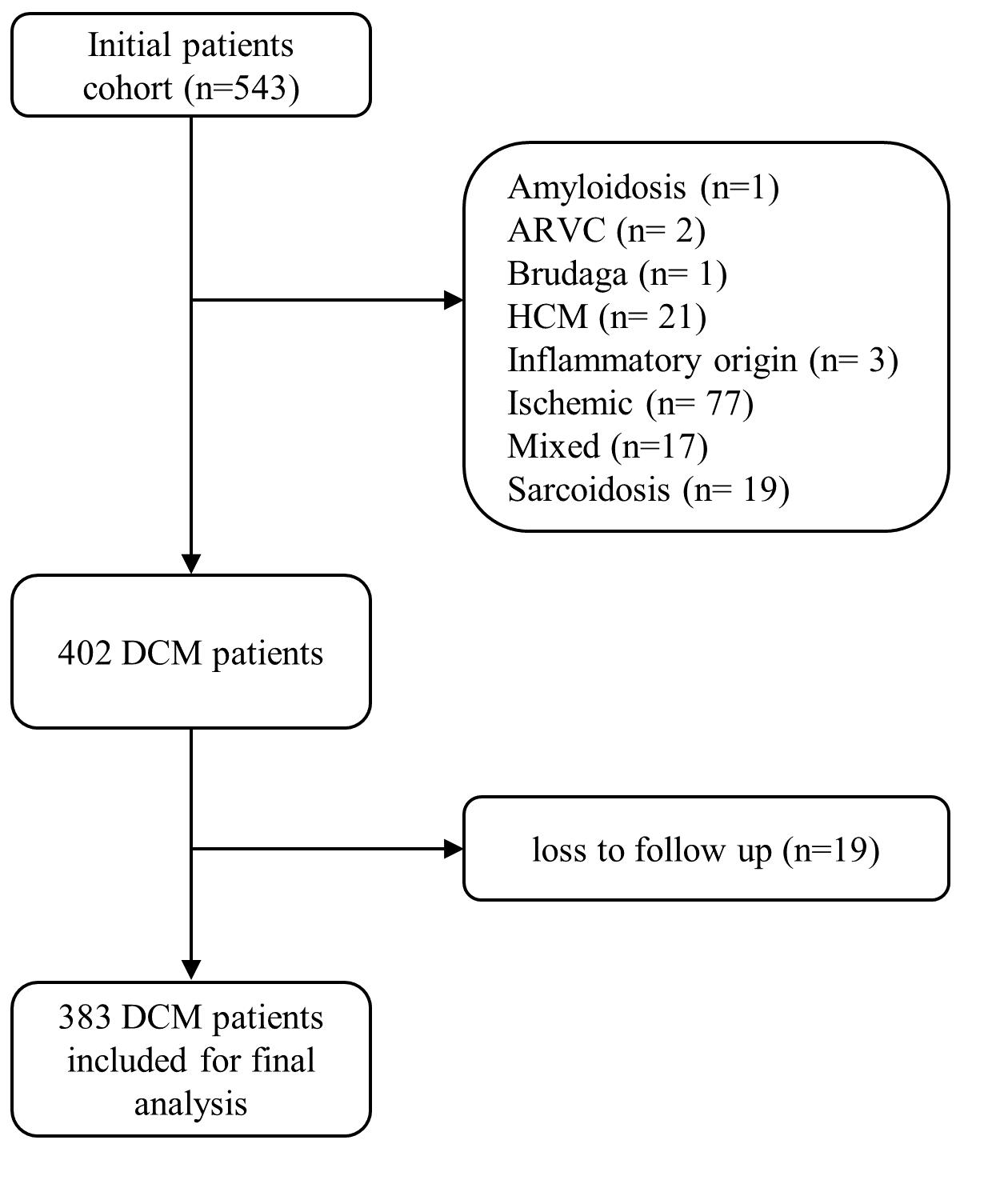


**Supplemental Figure 1.** Flow-chart of enrollment. Underlying etiologies were extracted from reviewing the established diagnoses noted in patients’ clinical records and determined by treating cardiologists based on our clinic's routine work-up, which includes invasive and non-invasive assessments as well as genetic tests. Abbreviations: DCM, dilated cardiomyopathy; HCM: hypertrophic cardiomyopathy; ARVC, arrhythmogenic right ventricular cardiomyopathy; LGE: late gadolinium enhancement.


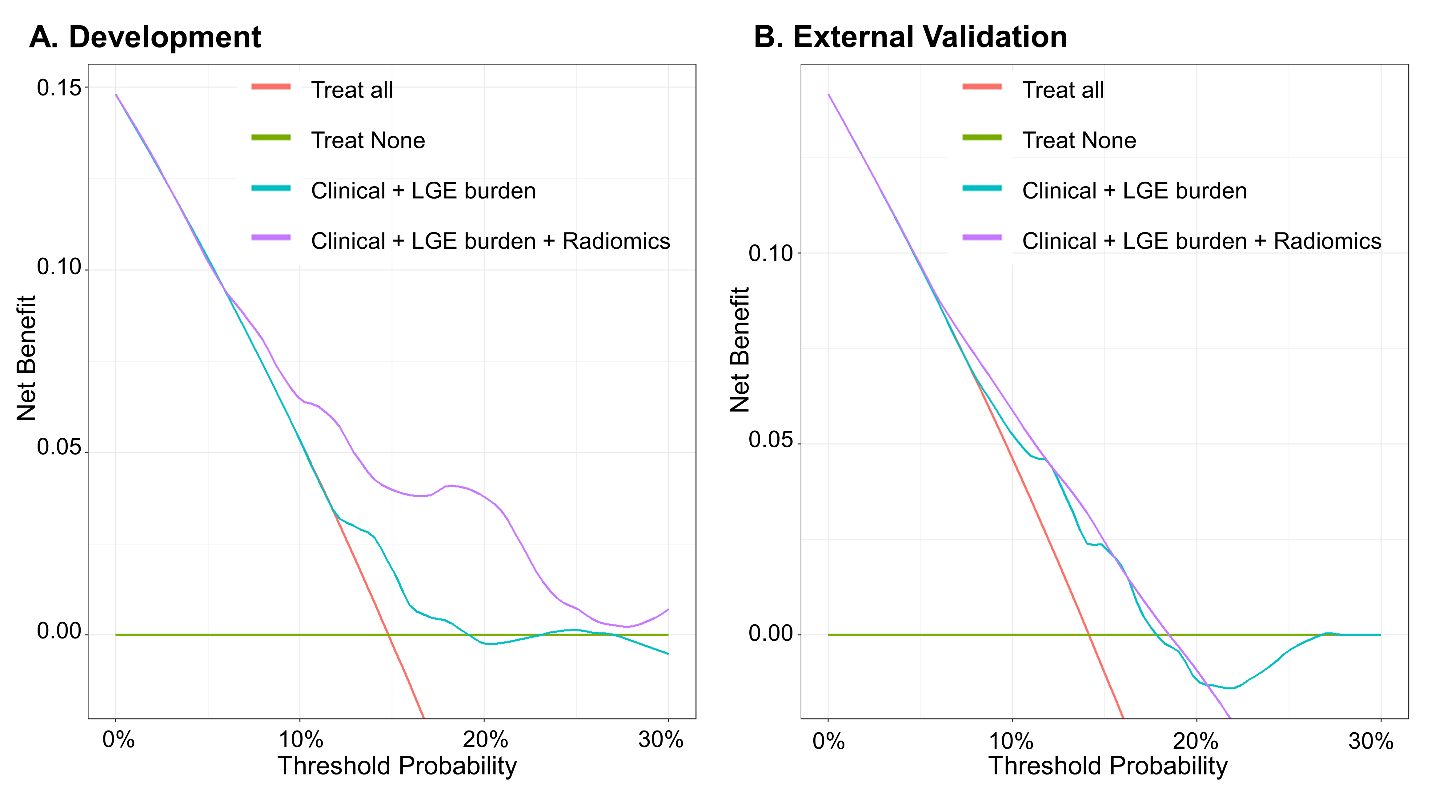


**Supplemental Figure 2.** Decision curve analysis for clinical + late gadolinium enhancement (LGE) burden and clinical + LGE + radiomics models for (A) development and (B) external validation cohorts.


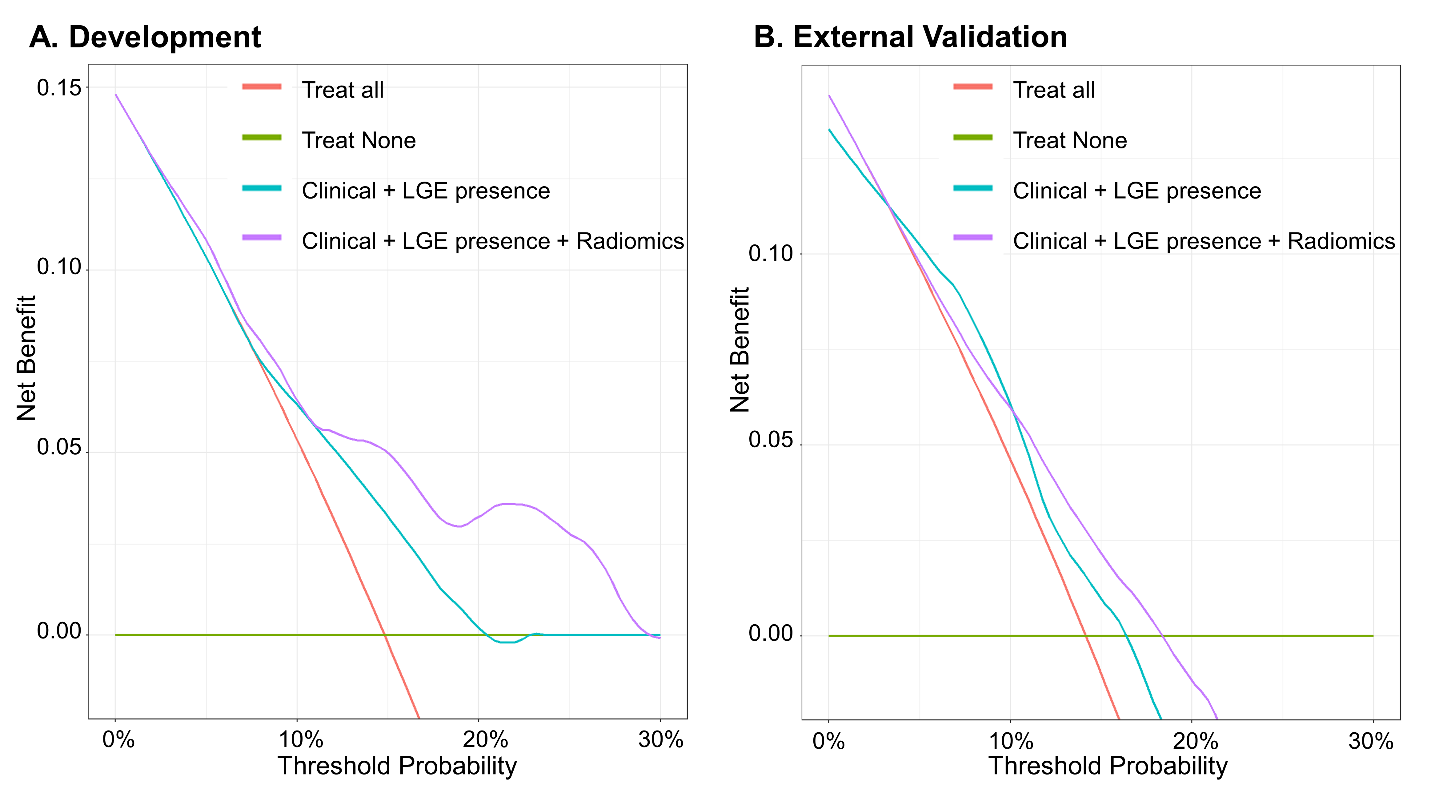


**Supplemental Figure 3.** Decision curve analysis for clinical + late gadolinium enhancement (LGE) presence and clinical + LGE + radiomics models for (A) development and (B) external validation cohorts.

**A. CMR Image Acquisition Parameters**

The imaging protocol included balanced steady-state free-precession cine sequence (slice thickness, 8-mm; gap, 2-mm, in-plane spatial resolution 2×2mm, 30 ms temporal resolution) and Late gadolinium enhancement. LGE images were acquired 10 to 20 min after injection of 0.1-0.2 mmol/kg of gadolinium-based contrast agents (Magnevist, MultiHance, Gadoterate Meglumine). In 258 patients in Cleveland Clinic Foundation, short-and long-axis 2D inversion recovery LGE images were acquired using a breath-hold, segmented inversion-recovery sequence (8-mm slice thickness, 10-mm inter-slice gap, TR, 6.4 ms; TE, 3.2 ms; FA, 25°; FOV, 320 × 320 mm^2^; and spatial resolution of 2 mm^2^). For the remaining cases, LGE was performed using a 3D inversion recovery sequence (TR, 5.3 ms; TE, 2.5 ms; FA, 25°; FOV, 320 × 320 × 100-125 mm^3^; spatial resolution, 1.5 × 1.5 × 3 mm^3^). In Beth Israel Deaconess Medical Center, short- and long-axis 2D inversion recovery LGE images were acquired using a breath-hold, segmented inversion-recovery sequence (8-mm slice thickness, 2-mm inter-slice gap, TR, 4.2 ms; TE, 1.8 ms; FA, 20°; FOV, 320 × 320 mm^2^; and spatial resolution of 2 mm^2^).

**B. Report on image biomarker extraction based on the image biomarker standardization initiative (IBSI) reporting guidelines.**

| **Patient** | | | |
| --- | --- | --- | --- |
| Region of interest | Myocardium | | |
| Patient preparation | Breath-hold instructions given to patients prior to image acquisition | | |
| Contrast agent | Late gadolinium enhancement (LGE) images were acquired 10 to 20 minutes after intravenous administration of 0.1-0.2 mmol/kg of gadolinium-based contrast agents (Magnevist, MultiHance, Gadoterate Meglumine) | | |
| Comorbidities | No comorbidities that effect imaging quality | | |
| **Acquisition** | | | |
| Acquisition protocol | Standard cardiac MRI imaging protocol was used | | |
| Scanner type | Achieva 1.5T, Ingenia 3T (Philips Healthcare, Best, Netherlands); Magnetom Vida 3T (Siemens Healthineers, Erlangen, Germany) | | |
| Imaging modality | MRI | | |
| Static/dynamic scans | Scans were static except for cine  No temporal modelling technique was used | | |
| Scanner calibration | Regular calibration according to manufacturer’s manual for preventive services | | |
| Patient instruction | Patients were given instructions for breath holding during acquisition | | |
| Anatomical motion correction | ECG-gated; Breath-hold | | |
| Scan duration | 45 min | | |
| RF coil | 16- and 32- channel cardiac coil | | |
| Magnetic field strength | 1.5 T and 3T | | |
| Scanning sequence | LGE (CCF) | | LGE (BIDMC) |
| Repetition time (ms) | 6.4 | | 4.2 |
| Echo time (ms) | 3.2 | | 1.8 |
| Echo train length |  | |  |
| Flip angle (deg) | 25 | | 25 |
| Acquisition type | Magnitude or phase sensitive inversion recovery | | Magnitude or phase sensitive inversion recovery |
| In-plane resolution (mm^2^) | 2mm | | 2mm |
| Image slice thickness (mm) | 8 | | 8 |
| Image slice spacing (mm) | 8 | | 10 |
| **Image Registration** | | | |
| Registration method | | None | |
| **Image processing – data conversion** | | | |
| Data conversions | | Conversion from dicom to matlab data file (exclude meta data while preserving resolution and intensity dynamic range of original images) | |
| **Image processing – post-acquisition processing** | | | |
| Anti-aliasing | | None | |
| Noise suppression | | None | |
| Non-uniformity correction | | None | |
| Image normalization | | Intensity normalization: minimum = 0, maximum = 1 | |
| Other post-acquisition processing methods | | None | |
| **Segmentation** | | | |
| Segmentation method | | Manually using commercially available software (cvi42 workstation Version 5.14.1 (Circle Cardiovascular Imaging Inc., Calgary, Canada) | |
| Conversion to mask | | MATLAB poly2mask | |
| **Image processing – image interpolation** | | | |
| Interpolation method | | SimpleITK sitkBSpline | |
| Voxel dimensions | | 1×1 mm^2^ | |
| **Image processing – ROI interpolation** | | | |
| Interpolation method | | SimpleITK sitkNearestNeighbor | |
| Partially masked voxels | | N/A | |
| **Image processing – re-segmentation** | | | |
| Re-segmentation methods | | None | |
| **Image processing – discretization** | | | |
| Discretization method | | None | |
| **Image processing – image transformation** | | | |
| Image filter | | Original, exponential, gradient, logarithmic, squaring, square-root, local binary pattern, and wavelet | |
| **Image biomarker computation** | | | |
| Biomarker set | | gray level co-occurrence matrix (GLCM), gray level run length matrix (GLRLM), gray level size zone matrix (GLSZM), gray level distance zone matrix (GLDZM), neighborhood gray tone difference matrix (NGTDM) | |
| Software availability | | PyRadiomics 3.0.1  Python 3.9  Scipy-stat 1.6.2  Scikit-learn (0.19.1)  Lifelines (0.28.0)  R version 4.2.2 | |
| **Image biomarker computation – texture parameters** | | | |
| Functional parameters of computing all Radiomic features | | Default values set by the PyRadiomics library. | |
